# Radiation-Associated Pemphigus Vulgaris in a Patient With Preceding Malignancy: Treatment With Rituximab as a Valuable Option

**DOI:** 10.3389/fimmu.2019.03116

**Published:** 2020-01-21

**Authors:** Franziska Schauer, Norito Ishii, Maja Mockenhaupt, Leena Bruckner-Tuderman, Takashi Hashimoto, Dimitra Kiritsi

**Affiliations:** ^1^Department of Dermatology, Medical Center - University of Freiburg, Faculty of Medicine, University of Freiburg, Freiburg, Germany; ^2^Department of Dermatology, Kurume University School of Medicine, Kurume, Japan; ^3^Institute of Cutaneous Cell Biology, Kurume University, Kurume, Japan; ^4^Department of Dermatology, Osaka City University Graduate School of Medicine, Osaka, Japan

**Keywords:** autoimmune blistering disorder, desmoglein, desmosome, desmosomal adhesion, radiation therapy

## Abstract

Pemphigus is a chronic autoimmune blistering disorder, characterized by (muco-)cutaneous erosions due to autoantibodies against desmoglein 3 and/or 1. Pemphigus induction might be associated with drugs, malignancy or radiation therapy (RT); the latter being only rarely described. A rigorous literature review revealed around 30 cases of RT-associated pemphigus, which had been primarily treated with topical and/or systemic steroids, in some cases also dapsone or few other immunosuppressive agents were given. The most common underlying cancer type was breast cancer. We here present a 63-year-old male patient, who was pre-treated with adjuvant RT for larynx carcinoma 3 months before admission. He developed extensive cutaneous, ocular, and oral erosions. Despite the clinical picture comparable to a paraneoplastic pemphigus, the diagnosis of pemphigus vulgaris of mucocutaneous type was established based on the direct immunofluorescence, showing positive cell surface IgG and discrete C3 deposits, with matching cell surface IgG pattern on monkey esophagus. Serum autoantibodies to desmoglein 1 and 3 were highly positive. No further autoantibodies were found, thus paraneoplastic pemphigus was excluded. The patient was treated with high dose prednisolone, partially given intravenously up to 2 mg/kg per day, as well as topical disinfectants and class IV steroid cream. To stabilize the disease rituximab 2 × 1,000 mg was given, leading to clinical and serological remission for up to 2 years now. We show that rituximab represents a good treatment option for the frequently treatment-refractory RT-associated pemphigus, a clinically and immunologically specific RT-induced skin disorder, resulting in long-term clinical, and serological remission.

## Introduction

The incidence of pemphigus has been estimated to be between 0.5 and 34 cases/million inhabitants/year (1), thus the disease is considered to be rare. As factors related to the induction of pemphigus, specific drugs and malignancy, but also radiation therapy (RT) have been proposed. Malignancy-associated pemphigus has an incidence of 5–11%, while malignancy-associated bullous pemphigoid has a reported incidence of 5.8–10.2% in retrospective studies (2, 3). As the underlying malignancy, lung cancer was most common in pemphigus, and gastric cancer in bullous pemphigoid (3). Cases of RT-associated pemphigus have only rarely been described in the literature (Table 1). In this study, we report the development of pemphigus vulgaris (PV) in a patient who received RT for cancer treatment and review the literature on RT-associated pemphigus. Search terms included irradiation, radiation, radiotherapy, and cobalt therapy. The data provided include age, gender, underlying malignancy, dosage of RT, time intervals between RT and the onset of pemphigus, immunofluorescence data, and the treatment regime. In our patient we show that treatment with rituximab induces long-term clinical and serological remission for over two and a half years.

**Table 1:** Patients with radiation-associated pemphigus described in the literature.

**No**	**Age**	**Diagnosis**	**Time from radiation to start of eruption**	**DIF (cell surface)**	**IIF (cell surface)**	**Neoplasm**	**Radiation characteristics**	**Localization** **(irradiated area, non-irradiated area, generalized)**	**Treatment**	**References**
1	63	PV	1 month	IgG	IgG	Hypopharynx carcinoma	LN cervical R II-IV, L III-IV 54 Gy, tumor bed, and LN cervical II 63,9 Gy	Irradiated area with generalized progression	Prednisolone 2 mg/kg/day, topical clobetasol propionate 0.05% ointment (body), followed by rituximab 2 × 1,000 mg	Current case
2	48	PV	3 months	+	na	Medullary breast cancer	100 Gy	Irradiated area with progression to non-irradiated area	Prednisone 150 mg/day	(4)
3	56	PV	1 year	na	na	Epidermoid bladder carcinoma	65 Gy	Generalized mucocutaneous type	Prednisone 60 mg/day	(5)
4	65	PV	<1 month	na	na	Breast cancer	n.a	Irradiated area with generalized progression	Prednisone 80 mg/day, later 120 mg/day, methotrexate 25 mg, then azathioprine 100 mg/day	(5)
5	70	PV	14 days	IgG, C3	+	Gastric lymphosarcoma	40 Gy	Irradiated area with generalized progression	Prednisone 120 mg/day	(6)
6	70	PV	4 months	+	+	Solar keratosis on the forehead	48 Gy	Irradiated area to non- irradiated area	Prednisone 100 mg/day	(7)
7	52	PV	3 weeks	na	na	Bronchial squamous cell carcinoma	40 Gy	Irradiated area with generalized progression	Methylprednisolone intravenously 1,250 mg 6 days, then 1 mg/kg BW and tapering 45 days	(8)
8	73	PV	3 months	+	+	Breast cancer	55 Gy	Irradiated area with generalized progression	Prednisone 50 mg/day	(9)
9	70	PF	1 month	+	+	Laryngeal squamous cell carcinoma	60 Gy	Irradiated area with progression to non-irradiated area	Prednisone 2 mg/kg/day, azathioprine 50 mg/day	(9)
10	54	PV	3 weeks	+	IgG	Lymphoma	38 Gy	Irradiated area with generalized progression	Prednisone 2 mg/kg/ day	(10)
11	77	PV	na	+	+	Basal cell carcinoma	60 Gy	Irradiated area with progression to non-irradiated	Prednisone 100 mg/day, dapsone 100 mg/day	(11)
12	45	PV	1 week	+	na	Breast cancer	68 Gy	Irradiated area	Prednisone 80 mg/day	(12)
13	61	PV	2 months	+	na	SCC of the lower lip	70 Gy	Irradiated area with progression to non-irradiated	Prednisone 1 mg/kg/day	(13)
14	54	PV	1 month	IgG, C3	IgG	SCC of the lung	59.4 Gy	Irradiated area with progression to non-irradiated	Methylprednisolone intravenously, then oral prednisone	(14)
15	73	PV	3 weeks	+	na	Epidermoid carcinoma	66 Gy	Irradiated area with progression to non-irradiated	Prednisone 1.5 mg/day, followed by rituximab 6 x 375 mg/m^2^	(15)
16	49	PV	4 weeks	+	na	Breast cancer	50 Gy	Non-irradiated area (mouth and esophagus)	Prednisone 60 mg/day, methotrexate 15 mg weekly	(16)
17	48	PV	6 months	na	+	Breast cancer (ductal carcinoma *in situ*)	Megavoltage radiation therapy−50 Gy	Irradiated area	Prednisone 100 mg/day, topical steroids	(17)
18	61	PV	1 month	IgG, C3	IgG (rabbit tongue)	Epidermoid carcinoma of the piriform sinus	70 Gy tumor bed, 54 Gy cercical area	Irradiated area with generalized progression	Methylprednisolone 1 g, followed by oral prednisolone 1.5 mg/kg/ day	(18)
19	47	PV	Within days (face), 6 months body	C4	IgG	Acinic cell carcinoma of the parotid gland	39, 6 Gy (cheek and chin)	Irradiated area with generalized progression	Prednisolone 1 mg/kg/day, azathioprine 150 mg/day and rituximab 2 × 375 mg/m^2^	(19)
20	58	PV	21 days	IgG, C3	+	Low grade infiltrating ductal carcinoma	Tumor area 65 Gy, subclavicular area (46 Gy), right internal mammary node chain (50 Gy)	Irradiated area with progression to non-irradiated area	Prednisone 2 mg/kg/day, MMF 2 g/day	(20)
21	84	PV	8 months	IgG	IgG	Breast cancer	60 Gy	Non-irradiated area (mouth)	Topical clobetasol propionate ointment, oral prednisolone 1 mg/kg/day, azathioprine 2.5 mg/kg/day	(21)
22	58	PV	14 months	IgG	+	Breast cancer (*in situ* ductal)	na	Irradiated area with progression to non-irradiated	Prednisone 60 mg/day, azathioprine 50 mg/day	(22)
23	68	PV	<1 month	+	na	Breast cancer	40 Gy	Irradiated area with generalized progression	Prednisolone, MMF	(23)
24	37	PF	<1 month	+	+	Malignant thymoma	30 Gy	Irradiated area with generalized progression (after sun exposure)	Methylprednisolone 12 mg, azathioprine 50 mg/day, dapsone 100 mg/day	(24)
25	92	PF	3 months	+	na	Breast cancer (*in situ* intraductal)	50,4 Gy	Irradiated area with generalized progression	na	(25)
26	70	PF	12 months	IgG, C3	IgG	Breast cancer	60 Gy	Irradiated area with progression to non-irradiated	Dapsone 100 mg/ day, topical clobetasol propionate ointment	(26)
27	59	PF	1 month	IgG, C3	na	Extramammary Paget disease	52.5 Gy	Irradiated area with progression to non-irradiated	Prednisolone 0.5 mg/kg/day	(27)
28	65	PF	2 months	IgG, C3	na	Breast cancer	50 Gy	Irradiated area	Prednisolone 30 mg/day	(28)
39	44	PF	<2 months	IgG and C3, focal staining C3d, and C4d	na	Breast cancer	na	Irradiated area with generalized progression	Oral prednisolone 1 mg/kg/day, topical hydrocortisone 2.5% ointment (face), clobetasol propionate 0.05% ointment (body), oral dapsone	(29)
30	66	PF	1 month	IgG, C3	na	Breast cancer (ductal carcinoma *in situ*)	50 Gy	Irradiated area with generalized progression	Oral triamcinolone 40 mg/day	(30)

*BW, body weight; C3, complement 3; C4, complement 4; CS, cell surface; PF, pemphigus foliaceus; PV, pemphigus vulgaris; MMF, mycophenolate mofetil; na, not available; +, positive (fluorescence pattern not described)*.

## Methods

### Immunofluorescence (IF) Studies

Direct immunofluorescence (DIF) and indirect immunofluorescence (IIF) were performed in the Department of Dermatology, Medical Center—University of Freiburg. The FITC-labeled antibodies were anti-human IgG, IgA, IgM, and C3c (Dako, Hamburg, Germany) at a dilution of 1:200, 1:50, 1:50 and 1:500, respectively. For IIF on monkey esophagus, patients' sera were diluted 1:10; secondary antibodies used were FITC-labeled anti-human IgG (Dako, Hamburg, Germany) at a dilution of 1:100.

### Immunoblotting Studies

Immunoblotting of normal human epidermal extracts (31, 32), the BP180 NC16a domain recombinant protein (33), the BP180 C-terminal domain recombinant protein (34), the concentrated culture supernatant of HaCaT cells (35), normal human dermal extract (36), and purified human laminin-332 for both IgG and/or IgA antibodies (37) were performed at the Kurume University and Osaka City University as previously described.

### Enzyme-Linked Immunosorbent Assays (ELISA) Studies

Commercially available IgG ELISAs of Dsg1 and Dsg3 (MESACUP, MBL, Nagoya, Japan) were conducted according to the manufacturer's instructions and measured by ELISA reader.

## Results

**Figure 1:**
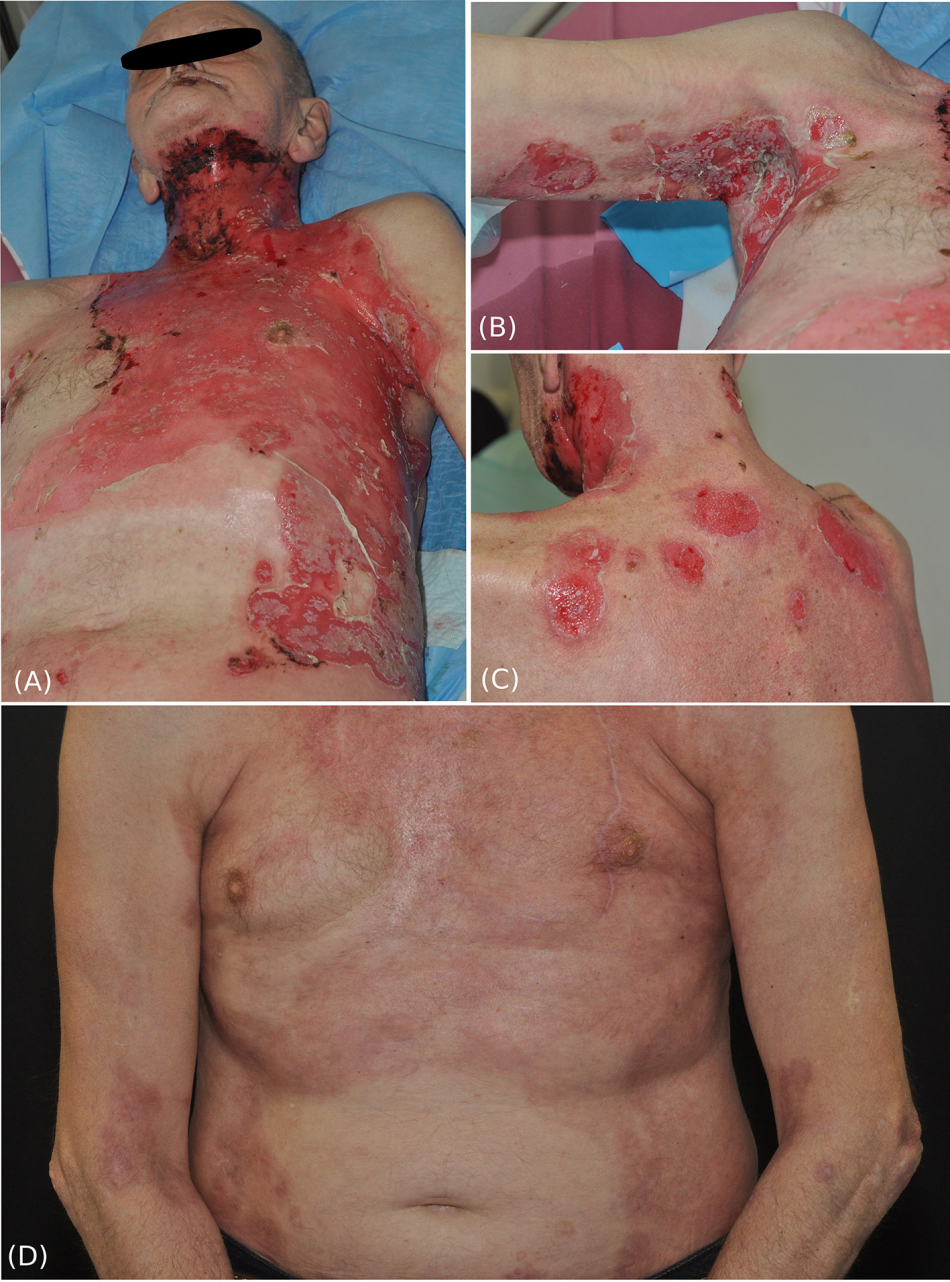
Clinical pictures of the patient at initial presentation and after treatment. Extensive erosive skin detachment of the frontal trunk and neck **(A)**, right axillary fold **(B)**, and shoulders and neck **(C)**. Three months after systemic treatment with rituximab and ongoing low dose glucocorticosteroids, intact skin with postinflammatory hyperpigmentations was observed **(D)**.

**Figure 2:**
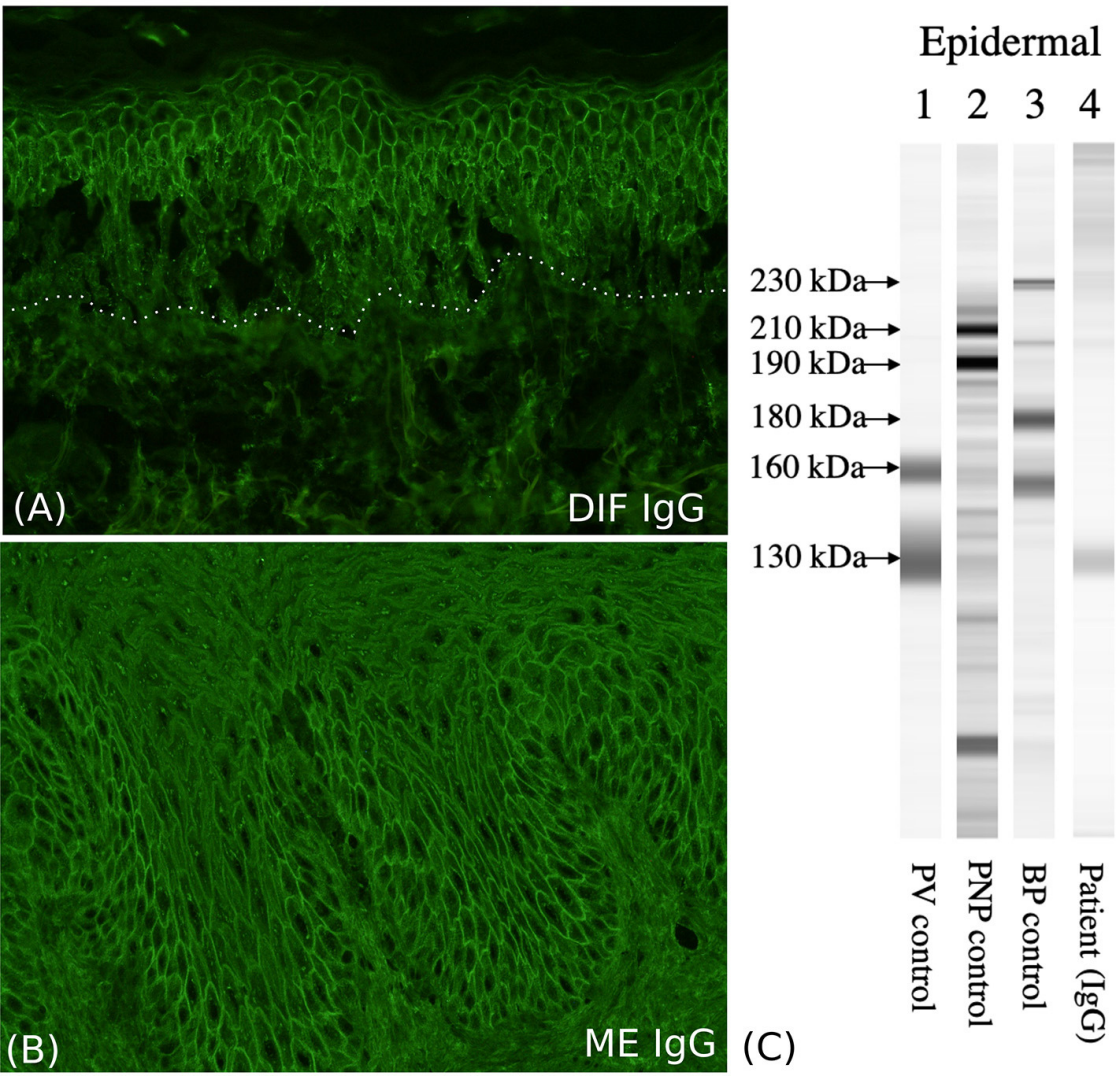
Immunological characterization of the patient. **(A)** The direct immunofluorescence (DIF) shows cell surface IgG deposition on the keratinocytes of the entire epidermis. **(B)** Indirect immunofluorescence (IIF) with monkey esophagus (ME) shows circulating cell surface IgG autoantibodies. **(C)** Immunoblotting with normal human epidermal extracts shows IgG antibodies reactive with 130-kDa Dsg3 (lane 4).

We present a 63-year-old male patient, who was referred to our department due to extensive cutaneous (Figures 1A–C) and mucosal erosions (eyes, nose, lips, mouth, and esophagus). No paronychia-like or lichen planus-like lesions were observed. Until 3 months prior to admission, the patient had received adjuvant RT, following surgical reconstruction and bilateral neck lymph node dissection to treat larynx carcinoma. At that time point, the patient had received 54 Gy (single dose 1.8 Gy each) for his cervical right (level II-IV) and left region (III-IV), as well as a single boost at the tumor bed and additional 63.9 Gy (single dose 2.13 Gy each) for the left cervical lymph nodes (level II). The tumor appeared to be in remission. Histopathology of a skin specimen showed an intraepidermal split with the characteristic tombstone pattern of the basal keratinocytes (not shown). DIF of a skin specimen from the thigh revealed cell surface deposition of IgG (Figure 2A) and C3 in the entire epidermis. IIF with normal human skin and monkey esophagus (Figure 2B) revealed circulating cell surface IgG autoantibodies at a titer of 1:160. No reaction to the basement membrane zone or transitional epithelium of rat bladder was observed.

Immunoblotting of normal human epidermal extracts demonstrated IgG autoantibodies against the 130-kDa Dsg3 (Figure 2C, lane 4) (32), but no reactivity with Dsg1, envoplakin, periplakin, BP230, BP180, LAD-1 antigen, collagen VII, or laminin-332. Other immunoblotting methods did not detect any other autoantigens, including BP180, LAD-1 antigen, collagen VII, laminin gamma-1, and laminin-332. ELISA results were positive for both anti-Dsg1 (191 units; positive index ≧ 20), and anti-Dsg3 antibodies (144 units; positive index ≧ 20). ELISAs for recombinant eukaryotic desmocollin 1-3 proteins were negative (38). The discrepancy between immunoblotting and ELISA for anti-Dsg1 antibodies can be explained by that the PV sera tend to react with non-conformational epitopes, which are destroyed during the procedure of immunoblot, but not ELISA. The higher sensitivity in ELISA diagnostics has been well-established by previous studies (39). In fact it has been shown that in immunoblot with normal epidermal extracts most PV sera react with Dsg3, while only about two-thirds or PF sera react with Dsg1 (32). PV with underlying malignancy and PNP are sometimes difficult to distinguish. However, although our case had severe oral and ocular mucosal paraneoplastic pemphigus-like lesions, immunoblotting showed reactivity neither with envoplakin nor periplakin, excluding the diagnosis of PNP.

Initially, the patient was treated with high dose prednisolone, first given intravenously up to 2 mg/kg per day, topical disinfectants, and class IV steroid creams, combined with meticulous wound care for several weeks. Administration of rituximab 2 × 1,000 mg led to quick stabilization of the disease and to clinical and serological remission. Currently, the follow-up-time is two and a half years (Figure 1D), showing that rituximab is a valuable treatment for this rare pemphigus subtype; it improved the patient's quality of life tremendously.

A rigorous literature search for RT-associated pemphigus revealed 29 reported cases (Table 1). Of these, 23 represented PV and eight pemphigus foliaceus (PF). The age of the patients ranged from 37 to 92 years, with a median of 62 years. In 11 cases, the disease onset was within <1 month, in 12 cases skin lesions developed within the first 3 months after therapy initiation. Twenty-seven of 30 (90%) cases initially presented with lesions at the irradiated side with the majority of these patients (24/30 cases, corresponding to 80%) having a progression to the non-irradiated skin (11/24 cases, corresponding to 46%) or even to a generalized distribution (13/24 cases, corresponding to 54%). The skin lesions stayed at the irradiation side in only three patients (3/30 cases, corresponding 10%). Two patients developed erosions in the non-irradiated area at their first presentation, while one patient showed a generalized distribution pattern of PV. At least three cases (including ours) clinically imitated PNP. The pemphigus diagnosis was made in 20 cases by DIF, and in 14 of these cases additionally by IIF. ELISA was performed in only four cases. Fifteen patients (*n* = 9 PV, *n* = 6 PF) were treated for breast cancer. Others received radiotherapy for miscellaneous forms of cancer, for example lymphoma, gastric lymphosarcoma, squamous cell carcinoma of the skin or the lung, and bladder carcinoma. The RT dose varied from 38 to 100 Gy, conducted in fractional application. All published patients, but one, have received oral, or intravenous glucocorticosteroids (prednisolone or methylprednisolone) in a medium to high dose. Ten individuals, representing one third of the reported, needed additional agents like dapsone, azathioprine or mycophenolate mofetil, at least temporarily. In two previously published cases rituximab had been given in different dosages (2 × 375 and 6 × 375 mg/m^2^) and resulted in remission with a follow up of up to 6 months (15, 19). Three patients had a pre-existing PF (27–29) that aggravated during radiotherapy; suggesting that in patients with a history of an autoimmune blistering skin disorder, RT should be cautiously considered. Nevertheless, it is not an absolute contraindication, since patients with PV have tolerated radiation without exacerbation of the disease (40).

## Discussion

Here we report a severely affected patient with PV in whom RT for larynx cancer triggered the onset of the disease. So far, 29 cases RT-associated pemphigus have been reported in the literature with the majority of cases having lesions first at the RT site, soon followed by generalized erosions (Table 1). This clinical sign facilitates differentiation from a classical RT-induced dermatitis. In our case, high dose prednisolone and B-cell depletion by rituximab was required to induce clinical and serological remission for more than two and a half years, without need for further immunosuppression. Since this is the third case of RT-associated PV treated with rituximab, showing a quick response and resulting in clear remission in all three cases, we propose that rituximab should be considered early, when treating such patients. Rituximab selectively binds to CD20 and leads to B-cell depletion due to antibody- and complement-dependent cytotoxicity, as well as induction of apoptosis. The use of immunosuppression in patients with malignancies should be avoided, whenever possible. However, in around one third of the already reported RT-associated pemphigus cases such drugs had to be given to control disease (Table 1). Thus, especially for the most severely affected patients, requiring additional immunosuppression than steroids, rituximab given at the already published regimen for PV (41) (1,000 mg rituximab on days 1 and 15) would be probably the most advantageous treatment option.

Another autoimmune skin blistering disorder that is pathogenetically often connected to malignancy or RT is bullous pemphigoid (BP). The so far published cases comprise similarly to RT-associated pemphigus more than 30 (42), including a stage IV melanoma patient with dual exposure to PD-1 checkpoint inhibition and RT therapy (43). Nguyen et al. have summarized 29 patients with BP following radiation for malignancy treatment. BP was localized on the irradiated area in 25 of them, in two it was localized in non-irradiated sites and in another two it was generalized (44). All cases had a rather benign course, with corticosteroids being sufficient to control the disease. RT-associated pemphigus appears to have a more severe disease course, since it tends to spread to non-irradiated skin and in one third of cases further treatment with other immunosuppressants was required to control disease.

Several hypotheses have been proposed about the role of RT in induction of autoimmune skin blistering disorders. The efficacy and role of RT in anti-tumor therapy is believed to be due to the RT-induced DNA damage to malignant cells, but recent evidence demonstrates that RT also activates the innate immune system, promoting specific danger signals like complement, calreticulin, and high mobility group box 1 protein (HMGB1), as well as a variety of cytokines and chemokines (45). Beside a few patients who may have circulating autoantibodies before RT, the substantial role of complement in activation of BP is well-established, while the role of complement in pemphigus has a controversial standing (46). HMGB1, released by damaged cells can stimulate macrophages and dendritic cells, thus resulting in an activation of T cells. Increased serological concentration, paired with plentiful cytoplasmic overexpression of HMGB1 and its receptor RAGE has been observed in the epidermis of pemphigus patients (47). This results in tissue destruction and unmasking of epidermal structures, thus promoting auto-reactivity in the irritated, immunocompromised area (48, 49). Furthermore, there are no sufficient data about the effects of RT damage and recovery of the thymus, which is in anatomical proximity to the tumor region in our patient. Post-RT effects in total body RT mouse model comprised reduced thymocyte numbers and long-term suppression of thymopoesis (50), supporting a hypothesis of impaired T-cell education and disturbed selection processes inducing autoimmunity. Finally, RT-associated pemphigus appears to have a similar incidence as pemphigoid. This contrasts the situation in the non-radiated population, where BP is more common. That is another argument for the direct induction of autoantibodies by the tumor in the RT-associated pemphigus. Additional studies to characterize the pathogenesis of RT-associated pemphigus are required to gain a better understanding of the disease.

Taken together, we show that rituximab represents a good treatment option for the frequently treatment-refractory RT-associated pemphigus, resulting in long-term clinical, and serological remission.

## Data Availability Statement

All datasets generated for this study are included in the article/supplementary material.

## Ethics Statement

The study has been approved by the Ethics committee in Freiburg (Number 235/15). All analyzes were performed with written informed consent of the patient, after ethics approval and in accordance to the Declaration of Helsinki.

## Author Contributions

FS and DK contributed conception and design of the study, they also wrote the initial manuscript draft. NI and TH performed part of the diagnostics and wrote sections of the manuscript. FS, MM, and DK cared for the patient, performed part of the diagnostics, and interpreted the results. LB-T interpreted the data and revised the manuscript critically for important intellectual content. All authors contributed to manuscript revision, read and approved the submitted version.

### Conflict of Interest

The authors declare that the research was conducted in the absence of any commercial or financial relationships that could be construed as a potential conflict of interest.

## Abbreviations

DIF: direct immunofluorescence
HMGB1: High mobility group box 1
IIF: indirect immunofluorescence
ELISA: Enzyme-linked immunosorbent assay
PF: pemphigus foliaceus
PNP: paraneoplastic pemphigus
PV: pemphigus vulgaris
RAGE: receptor for advanced glycation endproduct
RT: radiation therapy.
